# Comparison between inductively coupled plasma-mass spectrometry and benchtop X-ray fluorescence performance for trace elemental exposure in rat tissues

**DOI:** 10.1016/j.jtemin.2025.100229

**Published:** 2025-06

**Authors:** Kolawole E. Adesina, Stefano A. Parducci, Joseph D. Brain, Ramon M. Molina, Marc Weisskopf, Aaron J. Specht

**Affiliations:** a School of Health Sciences, Purdue University, West-Lafayette, IN 47906, USA; b Harvard T.H. Chan School of Public Health, Boston, MA 02115, USA

**Keywords:** Elemental contents, Icp-ms, Benchtop x-ray fluorescence, metals, organs, rat tissues

## Abstract

**Background:**

Trace elemental toxicants induce health detriment in almost every organ system in the human body and account for a large amount of environmental and ecological environmental pollution. Traditionally, inductively coupled plasma mass spectrometry (ICP-MS) has been the gold standard for measuring elemental concentrations in biological tissues collected from toxicological and epidemiological studies. However, ICP-MS is often limited by its complexity, cost, and time-intensive nature.

**Methods:**

This study investigates the feasibility of benchtop X-ray fluorescence (XRF) as an efficient alternative for trace elemental analysis in rat tissues, offering comparable quantification capabilities with enhanced operational simplicity. We conducted a comparative analysis using tissue samples from multiple rat organs, including stomach, eyes, and liver.

**Results:**

The elemental concentrations of Arsenic (As), Cadmium (Cd), Copper (Cu), Manganese (Mn), and Zinc (Zn) were measured using both ICP-MS and a high-powered benchtop XRF (Epsilon 4, Malvern Panalytical). Our findings demonstrated strong linear regression correlations between the two methods: As (R^2^ = 0.86), Cd (R^2^ = 0.81), Cu (R^2^ = 0.77), Mn (R^2^ = 0.88), and Zn (R^2^ = 0.74). The overall Pearson correlation coefficient was *r* = 0.95 (*p* ≤ 0.05), indicating high concordance between the mean concentrations obtained from ICP-MS and benchtop XRF. The median minimum detection limits for the elements were 0.12 μg/g, with specific limits for Cd (0.0042 μg/g), Cu (0.040 μg/g), Zn (0.12 μg/g), As (0.25 μg/g), and Mn (0.35 μg/g) over a 7.5-minute measurement period. Bland-Altman analysis revealed high agreement between the two methods, particularly for As, Cu, and Mn.

**Conclusion:**

These results suggest that both ICP-MS and benchtop XRF are viable for elemental quantification in organ tissues, with benchtop XRF being more practical for low-mass samples. This study shows benchtop XRF’s potential for high-throughput, accurate trace element analysis in biological samples, broadening its use in environmental and toxicological research.

**Synopsis:**

Human and ecological tissues of varying compositions and densities can be measured effectively using benchtop X-ray fluorescence

## Introduction

Analysis of elemental composition within the body offers valuable insights into their impact on health. Exposure to heavy metals in the environment has detrimental effects on neurological health, cardiovascular health, and nearly every system in the body [1–5]. Certain trace elements are vital for sustaining physiological and biochemical processes [6]. These minerals are essential recognized as essential trace elements due to their crucial role in ensuring health and wellbeing of both animals and humans [6,7]. An imbalance in these essential trace elements can lead to suboptimal growth and performance, poor health, and even increased mortality [6,8].

Toxicological studies are the primary driver for identification of how these toxic elements are affecting health in a controlled environment. For many years, various analytical atomic spectrometry techniques have been utilized to analyze and quantify essential trace elements in tissues and biological fluids, providing invaluable insights into elemental composition crucial for understanding biological processes and diagnosing health conditions [9]. For most toxicological studies, trace elements in biological samples are assessed using Inductively Coupled Plasma Mass Spectrometry (ICP-MS), Inductively Coupled Plasma Atomic Emission Spectrometry (ICP-AES), and/or Atomic Absorption Spectrometry (AAS) [10–15]. These methods, while effective, are considered “destructive” as they usually require sample digestion before analysis. They are time-consuming, relatively expensive, and demand significant technical expertise [16]. Both AAS and ICP-MS techniques need a considerable amount of time, with typical sample read times (~3–5 min) constituting only a small portion of the total time required for sample preparation, calibration standards (run between samples), and quality control steps, which collectively sum up to significant amount of time, extending the process to several days generally [17,18].

X-ray fluorescence (XRF) is emerging as a valuable method for elemental quantification. XRF identifies elements by using an X-ray source to excite atoms within a sample, causing the emission of characteristic X-rays specific to the elements present. These emitted X-rays are detected and analyzed to quantify the concentrations of individual elements in the sample. The energy dispersive X-ray fluorescence (ED-XRF) spectrometer captures X-rays across a wide range of energies simultaneously and generates output as a plot of intensity versus X-ray photon energy. The detected voltages are directly proportional to the incoming photon energies. In energy dispersive analysis, a solid-state detector is utilized, which generates a continuous distribution of pulses corresponding to the photon energies [19]. The detector is equipped with a specialized device that produces output pulses with heights proportional to the photon energy. To process these signals, a multi-channel analyzer is employed, converting the output pulses into a digital spectrum. This digital spectrum is then further processed to extract the analytical data needed for elemental quantification [20]. XRF presents advantages of being non-destructive, limited sample preparation time, low-cost per analysis, and not requiring specialized technical skills for its operation compared with ICP-MS [21].

The newer benchtop X-ray fluorescence (BXRF) offers a much higher power alternative to handheld (portable) counterparts, resulting in more precise and repeatable measurements with lower detection limits in the part per million (ppm) range [22,23]. BXRF systems offer much higher signal to noise and greater consistency in measurement variables, making them more reliable for analyzing multiple samples simultaneously [21]. Several XRF technologies such as portable XRF (PXRF) and other ED-XRF has previously been employed to analyze biological samples, such as whole blood, toenail, serum, water, liver, and other tissue samples, although generally with relatively high detection limits [21–32]. However, comparative studies assessing detection limits, accuracy, and precision of the BXRF technique in the determination of various metals and metalloids in biological matrices have been limited.

Benchtop XRF systems offer significant analytical advantages over portable XRF, particularly in laboratory-based biomonitoring and toxicological research. While PXRF is valued for its mobility and convenience in field applications, its lower power output limits sensitivity and detection capabilities. In contrast, BXRF systems, such as the one used in this study, incorporate higher-powered excitation sources, advanced detector resolution, and optimized analytical algorithms, enabling improved measurement precision, lower detection limits, and greater reproducibility [21,33]. These enhancements make BXRF particularly well-suited for analyzing complex biological matrices, where accuracy and sensitivity are critical for detecting trace metals. Furthermore, BXRF allows for batch analysis of multiple samples, increasing efficiency for high-throughput studies. Unlike PXRF, which may require less acquisition times and a trade-offs in sensitivity, BXRF provides reliable multi-element detection in a controlled setting, reducing external variability within a reasonable time that yields higher sensitivity. This capability is especially valuable in biological tissue analysis, where precise quantification of trace metals is essential for evaluating environmental exposures and toxicological impacts.

ICP-MS is a robust and highly sensitive technique for elemental analysis; however, it is not without limitations. Samples analyzed using ICP-MS must be transported to a laboratory, where they undergo acid digestion, resulting in sample destruction and the generation of secondary hazardous waste. Furthermore, meticulous operation is required to avoid issues such as contamination, drift, and interferences, all of which can impact the accuracy and precision of the results [22]. In contrast, the new BXRF offers significant advantages over ICP-MS, including being non-destructive which preserves valuable biological samples for future analyses, requiring minimal sample preparation, and allowing faster analysis without the need for specialized technical expertise. Although previous research have used portable (handheld) XRF to measure elements like copper, zinc, and lead in liver tissues [9,34], cadmium, mercury and lead in kidneys [35,36], iodine in the thyroid, and iron in the skin, arsenic in soft tissue phantoms [37]. These studies validate XRF as an adoptable technique for elemental analysis across various human and animal tissues [38–41]. Given these capabilities, benchtop XRF should be well-suited to measure tissues of varying weights with detection efficiencies comparable to those of the gold-standard ICP-MS measures that has been carried out before. In this study, two multi-elemental analytical techniques, ICP-MS and BXRF, were employed to analyze trace elemental composition in rat tissues obtained from several organs including lungs, stomach, muscle, bone, eyes, liver.

This study presents a quantitative approach to assess the analytical performance of benchtop XRF and ICP-MS techniques for accurately quantifying trace elemental contents (arsenic, cadmium, copper, manganese, and zinc) by comparing the concentration of these elements in rat tissue samples obtained from different organs. This comparison presents a first study on the quantification of elemental contents in a variety of tissues applying the benchtop energy-dispersive XRF analytical method and aims at establishing the limits of detection and limitations of benchtop XRF for uses in toxicological and epidemiological sample analyses.

## Methods

### Rat organs and tissues preparation

The protocols used in this quantification study were approved by the Harvard Medical Area Animal Care and Use Committee. Thirty-seven Wistar Han male rats (8 weeks old at arrival) from Charles River Labs (MA) were used for this study. Rats were acclimatized to the animal facility environment for one week prior to experimental procedures. Rats were treated with intraperitoneal injection of either normal saline (*n* = 17) or zinc [(1 mg/kg Zn, *n* = 4), (5 mg/kg Zn, *n* = 12) or (10 mg/kg Zn, *n* = 4) in the form of either zinc sulfate or zinc gluconate for 3 consecutive days. Two days after the last injection, rats were anesthetized using vaporized isoflurane, the abdomen of each rat was opened, and blood collected from the posterior vena cava into EDTA or plain collection tube. Plasma and red blood cells in EDTA-treated blood, and serum sample from coagulated blood was also collected after centrifugation. Samples of lungs, liver, eyes, brain, testes, kidneys, heart, pancreas, prostate gland, along with blood, skeletal muscle, femur (hard bone), bone marrow (extracted from 2 femurs), fat, and skin were collected and placed into pre-weighed tubes. The gastrointestinal tract was partitioned into sections comprising the stomach, small intestine, cecum, remaining large intestine, and rectum and were also collected into pre-weighed tubes. Each sample weight was recorded. Samples weights ranged from 0.008 – 0.755 grams (g) and an average mean value of 0.312 (g).

### Benchtop X- ray fluorescence (XRF)

The Epsilon 4 benchtop X-ray fluorescence device, which is energy dispersive and manufactured by Malvern Panalytical in Marlborough, MA, was employed for this study. This equipment operates using a 15 watt metal ceramic X-ray tube with a silver (Ag) anode, with a maximum voltage of 50 kV. Similar to standard XRF practices, the Epsilon 4 software employs standard deconvolution protocols involving a sequence of Gaussian combines with a polynomial background. These protocols aid in identifying the net counts linked to each specific element of interest [29,42–44]. Tissue samples were positioned within the center of a 30 mm XRF circular cup and then pinned down using a 4-micrometer thin polypropylene film.

**Fig. 1.**
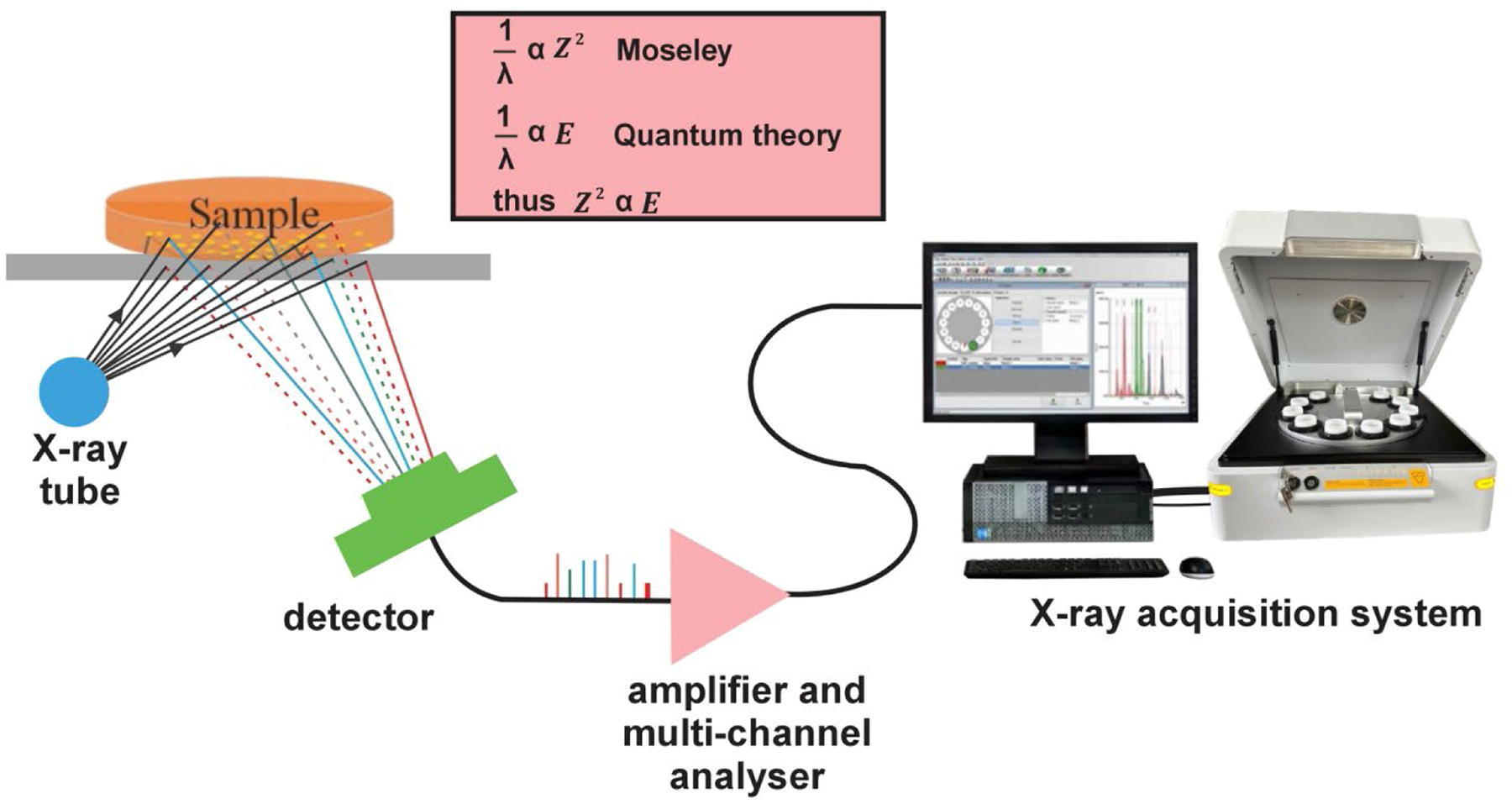
Schematics of benchtop energy dispersive X-ray spectrometer setup for multi-elemental detection and analysis in tissue samples.

In our BXRF analysis, there was no digestion of wet tissue samples or additional preparation post-cleaning [25]. To enhance accuracy and reliability, readings were taken with the sample slowly rotating during each measurement. This method addresses potential inhomogeneity, ensuring measurements that are more representative by accounting for localized variations in elemental distribution. While assessing the trace metal levels, we used one primary setting of the X-ray tube at 50 kV and 300 μA, collating the data for a duration of 7.5 min for a single tissue sample. Of note, the beam can be optimized for measurement of each element more distinctly, but this study was focused on those elements able to be quantified simultaneously. In BXRF, all sample elements present undergo simultaneous excitation, and the energy dispersive detector combined with multi-channel analyzer concurrently collects the emitted fluorescence radiation from the sample and separates the distinct energies of the characteristic X-ray emitted by each element present in the sample as shown in Fig. 1.

To calibrate the benchtop XRF device, we used 20 rat tissue samples as standards for other rat tissues. [23] Tissue-specific calibration was critical, as biological matrices exhibit variability in composition, density, and elemental distribution, which can influence the accuracy of measurements and needs to be accounted for. We aimed to identify if calibration from measured materials could be accurately done without a prior identification of the concentrations in calibration standards. These standards were analyzed using BXRF to obtain reference concentrations, which were then used to establish calibration curves for each element of interest.

Signal data was normalized in our benchtop XRF method using the Compton scattering peak, which is effective in addressing mass and thickness variations in biological samples. The Epsilon software allows for this normalization to be implemented alongside the deconvolution obtaining elemental signals [23,42,44]. This allowed us to identify the accuracy of the normalization technique over tissues from different organs with different masses. The signal from each of these samples was translated to concentration using the obtained ICP-MS values obtained later. To validate the reliability of the calibration method, calibration curves were assessed for linearity and precision using the root mean square error (RMSE). The calibration was further tested for reproducibility by analyzing multiple samples from different tissue types (e.g., liver, lungs, muscle, and bone) under identical measurement conditions. The consistency of results across tissues confirmed the reliability of the method in accounting for matrix effects and tissue-specific variability. This approach ensures that the system is not biased toward any single tissue type, making it broadly applicable for biological samples. The final results obtained from the benchtop XRF calibration are expressed in μg/g of tissue material, consistent with the standard units used in the ICP-MS analysis of our calibration tissues. In our analysis, we incorporated negative XRF measurement values in the correlation analysis with ICP-MS results to fully assess the performance and limitations of the XRF method and recognizing them as point estimates of concentration prone to measurement error. This approach ensures a comprehensive comparison, reflecting the true detection capabilities of both methods and providing a more accurate analysis of elemental quantification in biological samples. Excluding these measurements below the detection limit might falsely alter correlations due to decreased variability [45–47].

The selection of elements for further normalization and analysis was based on their significant roles in environmental toxicology and human health. The specific element results we particularly focused on in this work were arsenic (As), cadmium (Cd), copper (Cu), manganese (Mn), and zinc (Zn). As and Cd, commonly utilized in commercial applications, give rise to environmental issues that could ultimately pose a threat to human health [48,49]. It has also been previously observed that exposure of rats to Cd leads to an increase in Cu levels in the kidney, but this effect have been attributed to the accumulation of Cd in the kidney in the form of a complex copper-containing metallothionein [50–52]. Of which, metallothionein, a cytoplasmic protein with a low molecular weight and a high cysteine content, is primarily synthesized in the liver and kidneys in response to treatment with Zn, Cu, and Cd in animals [53]. Mn accumulation in the brain leads to toxic effects and has been linked to acquired hepatocerebral degeneration. This condition is commonly associated with chronic liver disease, and liver transplantation remains the most effective treatment for mitigating the degenerative effects caused by this illness. On the other hand, Zn plays a critical role in various aspects of human metabolism but its insufficiency is also associated with weakened immune responses and reduced ability to combat harmful external agents, highlighting the importance of addressing this deficiency to support overall health and metabolic function [54]. Furthermore, research into prenatal nutrition highlights the importance of these essential trace elements, including Cu, Mn, and Zn, for the healthy development of fetuses, underscoring their vital role in ensuring proper growth and future well-being. Therefore, the health risk posed by trace elements in the environment, which can be absorbed into the food chain by animals and humans, may lead to morphological abnormalities and genetic alterations in cells and impact enzymatic and hormonal activities [55]. Hence, the motivation for this study, to assess the levels of these trace elemental contaminations in animal and humans, by employing the appropriate instrumentation for this purpose.

### Inductively coupled plasma mass spectrometry (ICP-MS) measurements

The tissue samples underwent digestion using 800 μL of concentrated nitric acid for a duration of 12 h at 80 °C. Subsequently, the resulting digests were diluted to achieve a total volume of 40 mL with ultrapure water and then filtered through a 0.22 μM PES syringe filter. The filtered solution was directly subjected to measurement done with internal standards *Sc*, Y, and Tb using ICP-MS at Luna Nanotech (Ontario, ON, Canada). All sample weights were recorded. Each sample underwent measurement to determine the content of As, Cd, Cu, Mn, and Zn. The final data were presented both as μg/g, indicating the proportion of the element in each organ tissue.

### Limits of detection (LOD) of benchtop X-ray fluorescence

The limit of detection (LOD) for a specific element relies on the net counts within the spectral line of that element, compared to the standard deviation of the background counts within the same energy interval [56–58]. LOD is determined as the measurement condition in which the counts within the specific spectral line are at two times greater than the standard deviation of the background counts [56]. It also shows an inverse relationship with the square root of the measuring time; in other words, a fourfold rise in measuring time reduces the LOD by a factor of two [58]. The LOD is expressed as the minimum concentration of an analyte that an instrument can consistently and dependably detect [59,60]. This is the lowest concentration of any element that the method can detect but in return usually decrease the accuracy in great amount. The limits of detection (LOD) are calculated using the formula below;

(1) $$\text{LOD}=2\times \frac{\sigma_{0}}{\text{m}}$$

Where *σ*_0_ is the standard deviation of the signal (square root of counts at 0 ppm) and m is the slope of the regression between counts and ppm taken from our rat tissues measurements [30]. Employing actual tissues to set the detections allows for accommodating any probable uncertainty that might arise due to inherent instrumentation repeatability errors when measuring the sample itself. To establish this detection limit inclusive of instrument error, tissue samples underwent 30 repeated measurements employing the same X-ray setting. The LOD was determined as twice the quantified element standard deviation over these 30 measurements.

The formula (Eq. (1)) assumes that the XRF system produces a linear relationship between the measured signal intensity and elemental concentrations. Additionally, it presumes that the background signal remains stable and consistent across all measurements, with minimal noise interference in the spectral region of interest. For our specific study, several assumptions were made regarding the LOD calculation. First, the variability in tissue types was addressed by applying Compton normalization to account for differences in sample mass and geometry. In practice, differences in tissue density and matrix composition could affect the signal-to-noise ratio, potentially impacting the accuracy of LOD values for certain elements. Additionally, the LOD calculation relies on the assumption that each sample is homogeneous in elemental distribution. However, biological tissues are inherently heterogeneous, with elements potentially distributed unevenly at the microscopic level. This could lead to localized variations in detected signal intensities, particularly for low-concentration elements near the detection threshold. Another limitation arises from the potential spectral interferences caused by overlapping peaks in complex biological samples. For example, the presence of neighboring elements with overlapping X-ray emission lines could increase background noise and reduce sensitivity for specific trace elements, such as As and Cd. Furthermore, elemental binding to biomolecules may alter local concentrations, affecting fluorescence yield and detection thresholds. Variations in sample hydration can also affect X-ray absorption, leading to subtle shifts in signal intensity. Additionally, radiation scatter within biological matrices can introduce measurement deviations, particularly in samples of varying thicknesses. All of these aforementioned effects were effectively addressed through our calibration model, which integrates normalization techniques to minimize their impact. Additionally, the system’s peak deconvolution algorithms further refine our measurements, ensuring the reliability of benchtop XRF for precise elemental quantification in complex tissues.

### Statistical analysis

The linear regression models and Pearson correlation coefficients were used to compare the concentration results obtained from both analytical methods, while Bland-Altman analysis was employed to determine the mean bias and limits of agreement between the two methods. Linear regression models (R^2^) validated the normal distribution of variables to estimate the beta, intercept, and variance at the 95 % confidence interval. All obtained values were included in the statistical analyses. Metal concentrations in tissue samples were individually correlated in scatter plots for both instruments. Regression analysis included calculating correlation coefficients using the least squares method, prediction limits, standard error of the intercept, and the significance of the deviation of the intercept from zero [61]. Pearson’s correlation coefficients (r) were also done in *R* to assess the linearity between the concentrations measured by ICP-MS and BXRF, evaluating the deviation from the ideal 1:1 relationship. It is important to note that the correlation coefficient does not directly imply agreement between the methods [62].

Bland–Altman analysis was employed to compare ICP-MS and BXRF methods by calculating the mean difference (bias) and establishing a 95 % confidence interval (CI) within which the differences between the two methods would fall, thereby constructing the limits of agreement. For each pair of measurements, y_1_ is the value from the first method and y_2_ from the second method.

The mean value was calculated as $\frac{\left(y_{1}-y_{2}\right)}{2}$ and the difference as (y_1_ - y_2_) [62–64]. These differences are then plotted against the corresponding mean values for all sets of measurements using the Eq. (2) below:

(2) $$\bar{y}=\frac{1}{n}\sum_{k=1}^{n}{\left(y_{1}-y_{2}\right)}_{k}$$

The 95 % limits of agreement are found as the mean bias ±1.96 times the standard deviation (SD) of the differences. If the expected differences fit within these limits, the two methods can be seen as interchangeable. This analysis relies on the assumption that the differences between the values from the two methods follow a normal distribution. For benchtop XRF calibration, we calculated the root mean square error (RMSE), representing the standard deviation of the calibration regression residuals in μg/g. To assess the effect of tissue sample weights on metal concentration results, we used ANOVA analysis using IBM SPSS statistics 27. All other statistical analyses and visualizations were carried out using *Python* (version 3.11.11) through Google Colab.

## Results

### Benchtop XRF energy spectrum in tissues

**Fig. 2.**
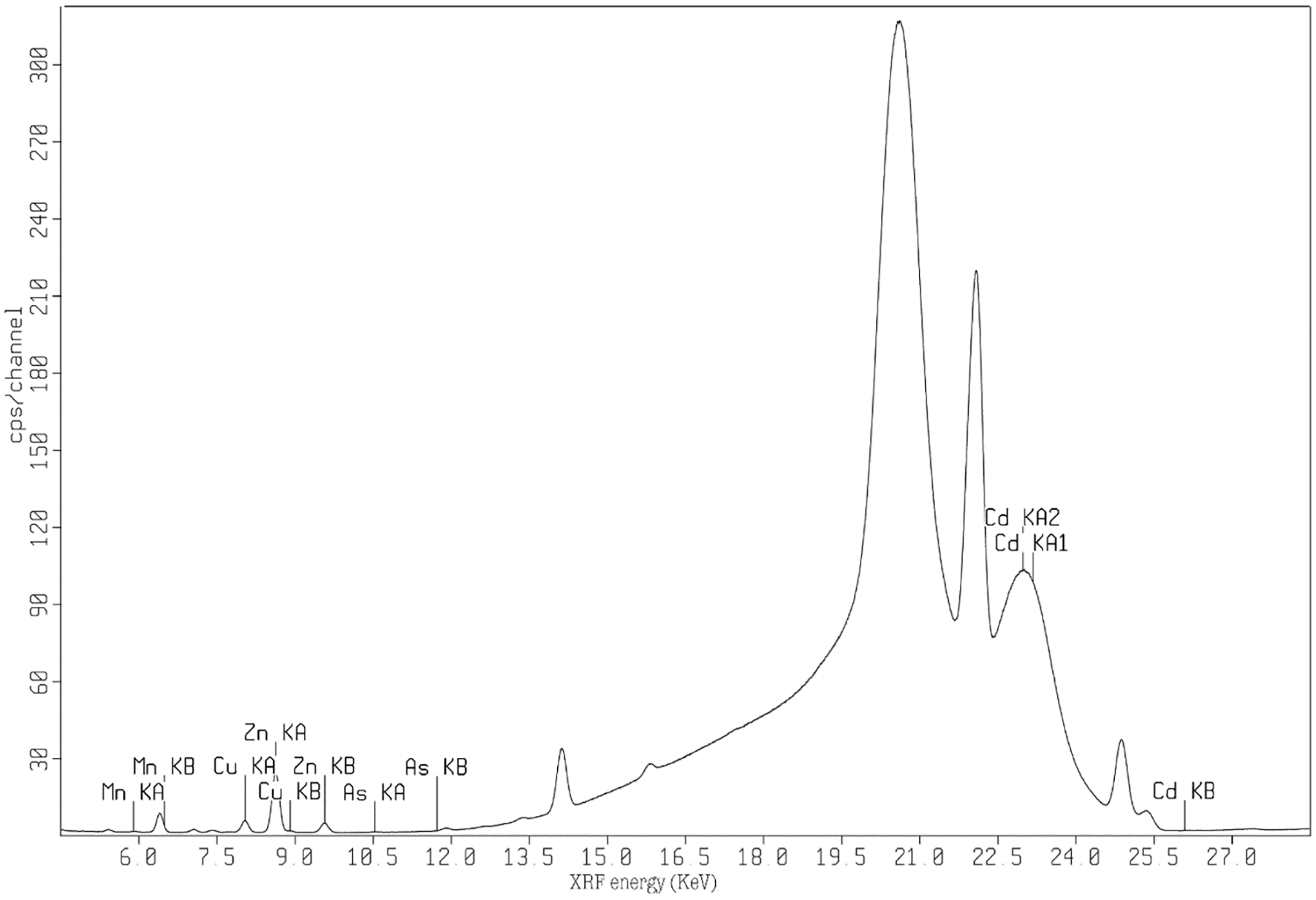
Benchtop XRF energy spectra for rat tissue standards trace elemental measurement.

Fig. 2 shows the established *ex-vivo* energy dispersive benchtop XRF spectrum obtained rat tissue samples. The summary of the analysis for the reference tissue standards including the range of elemental levels, root mean square equivalent (RMSE), and limit of detections are reported in Table 1.

### Benchtop XRF system calibration using standards

Goodness of fit parameters for benchtop XRF calibrations were conducted for As, Cd, Cu, Mn, and Zn in rat tissues. Table 1 presents the calibration performance of benchtop XRF for trace element quantification in rat tissues (μg/g, dry weight), highlighting calibration coefficients (R^2^), mean detection values, variation coefficients, and root mean square errors. The reference standard ranges illustrate the system’s accuracy across varying elemental concentrations, reinforcing the reliability of benchtop XRF for precise biological sample analysis. Here, the reference standards used are actual rat tissue samples as standards, ensuring accurate and appropriate calibration for measuring other tissue samples.

### Elemental content quantification and detection limits of bxrf and ICP-MS

**Fig. 3.**
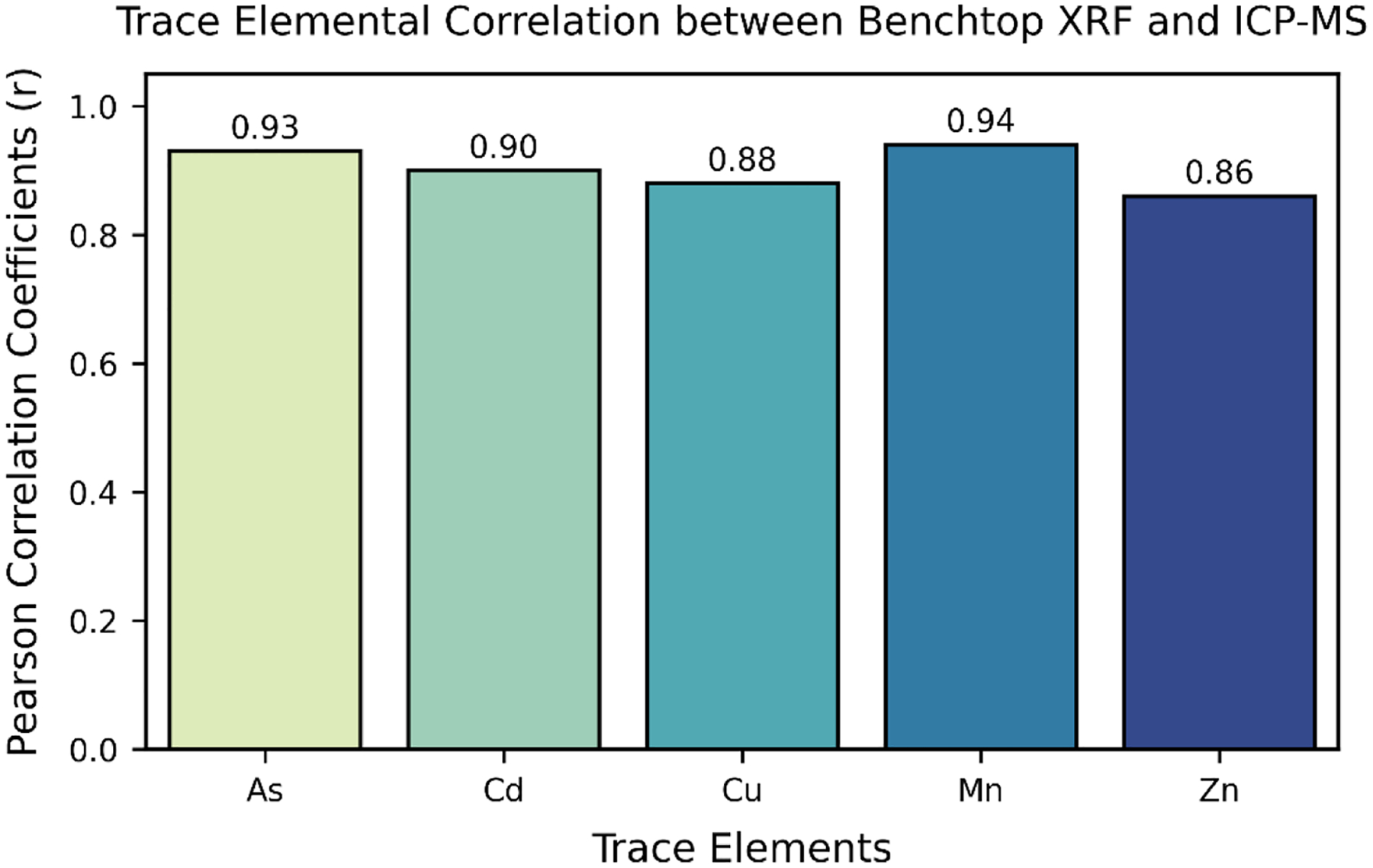
Pearson correlation coefficients between benchtop XRF and ICP-MS for trace elements measured in tissues.

Content of various trace elements measured from tissues obtained from dissected rat organs including the serum, lungs, bone, testes, stomach, heart, liver, eyes, and brain have already been reported in Table 2 as well as the detection limits of each element using the analytical methods of BXRF and ICP-MS (Fig. 3). The total mean elemental concentrations and detection limits (μg/g) measured in each tissue using benchtop XRF and ICP-MS are presented in Table 2.

### Correlation between tissue trace element levels

Strong and significant Pearson correlations were observed between the benchtop XRF and ICP-MS measurements (Fig. 3). Each element determined using both analytical instruments were examined for linearity, correlation, and its confidence interval. As gave Pearson correlation (*r* = 0.93, *p* < 0.05) in 123 tissue samples and RMSE of 0.0044 at beta (95 % CI = 0.017 [0.21, 0.240]). Cd gave Pearson correlation (*r* = 0.90, *p* < 0.05) in 104 tissue samples and RMSE of 0.0016 at beta (95 % CI = 0.00013 [0.00122, 0.0015]). Cu gave Pearson correlation (*r* = 0.88, *p* < 0.05) in 81 tissue samples and RMSE of 0.33 at beta (95 % CI = 0.11 [0.80, 1.026]). Mn gave Pearson correlation (*r* = 0.94, *p* < 0.05) in 79 tissue samples and RMSE of 0.073 at beta (95 % CI = 0.026 [0.28, 0.33]. Lastly, Zn gave Pearson correlation (*r* = 0.86, *p* < 0.05) in 184 tissue samples and at beta (95 % CI = 0.024 [0.25, 0.29]).

### Trend analysis of each trace element using benchtop xrf and ICP-MS

**Fig. 4.**
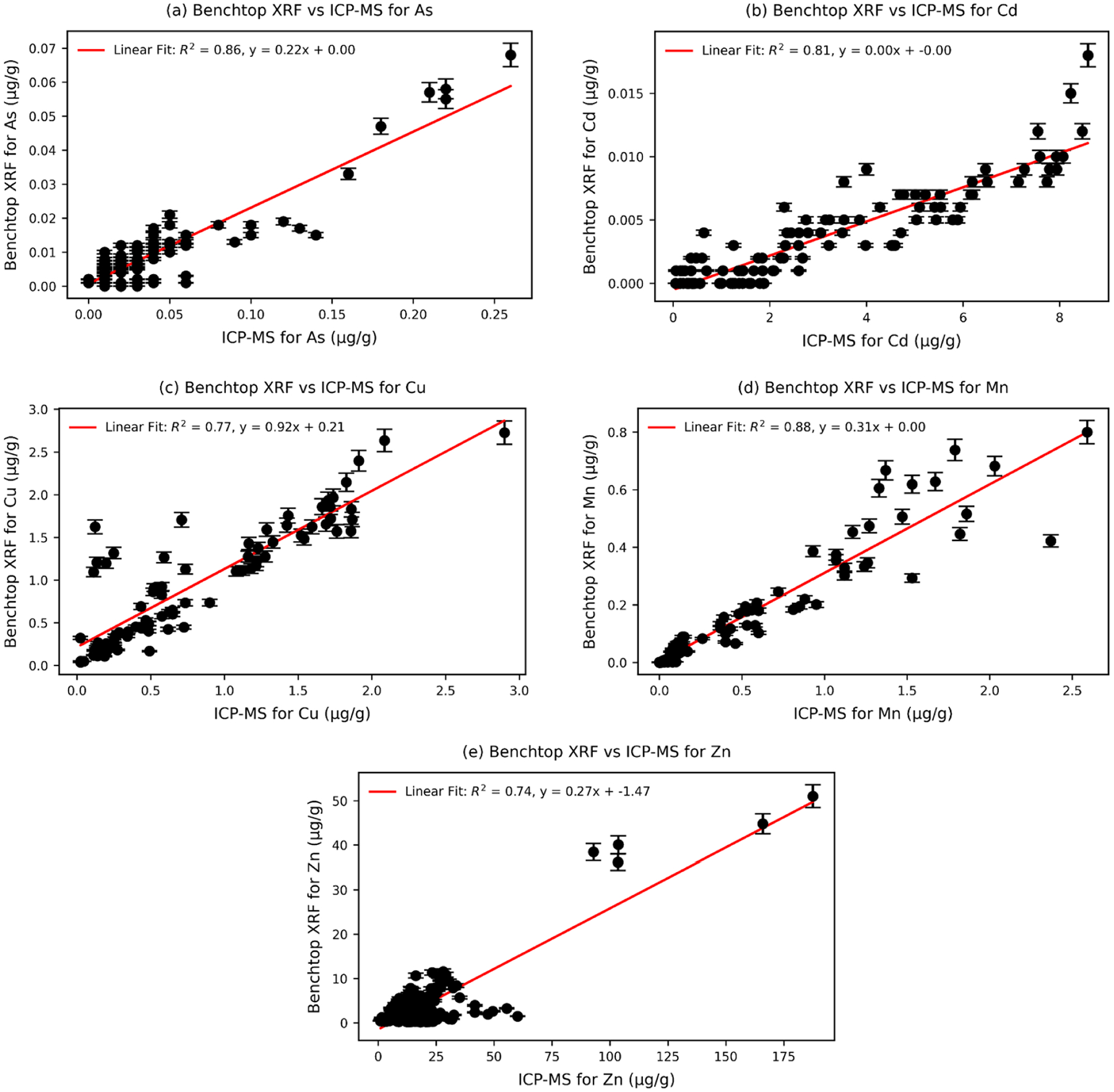
Bivariate analysis (scatter plots with regression lines) comparing trace elemental concentrations (μg/g) in rat tissue samples measured by benchtop XRF and ICP-MS for (a) As (*n* = 123, *r* = 0.93), (b) Cd (*n* = 104, *r* = 0.90), (c) Cu (*n* = 81, *r* = 0.88), (d) Mn (*n* = 79, *r* = 0.94), and (e) Zn (*n* = 184, *r* = 0.86). The x-axis represents concentrations determined by ICP-MS, while the y-axis shows corresponding values from benchtop XRF. The red lines indicate linear regressions, with R^2^ values representing the correlation strength. Black error bars indicate measurement uncertainties for both techniques.

Linear regressions were performed to compare the amount of trace element per gram of the tissue with the total trace element in each sample and all metals assessed in this study. The regression analysis showed strong coefficients of determination for all elements measured with benchtop XRF device and gave R^2^ = 0.86 for As, R^2^ = 0.81 for Cd, R^2^ = 0.77 for Cu, R^2^ = 0.88 for Mn, and R^2^ = 0.74 for Zn (Fig. 4). Fig. 4 shows the bivariate correlated data for both analytical techniques displayed for each element.

**Fig. 5.**
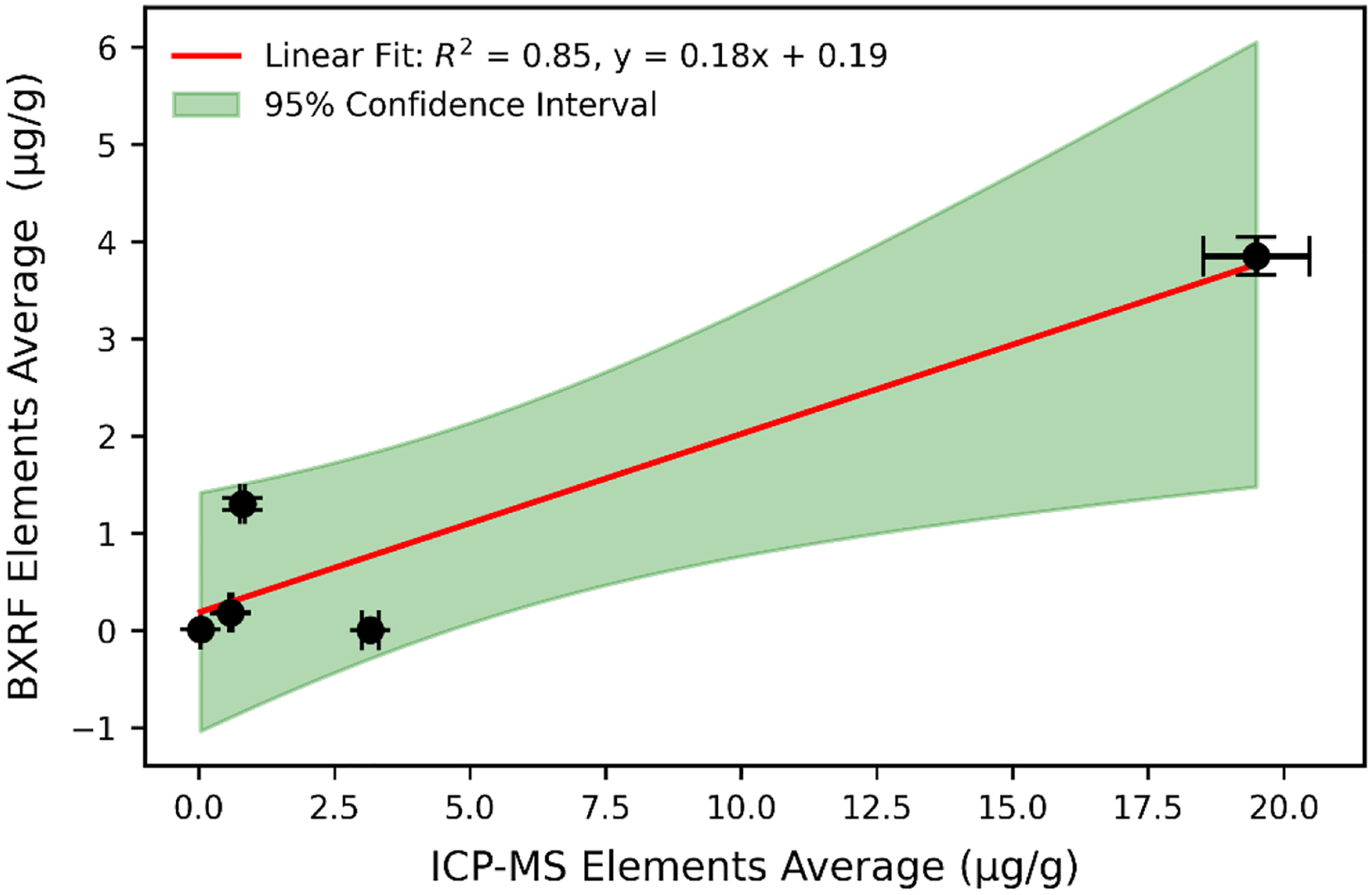
Linear regression analysis comparing elemental concentrations (μg/g) measured by benchtop XRF and ICP-MS. The x-axis represents mean elemental concentrations from ICP-MS, while the y-axis shows corresponding values from benchtop XRF. The red line represents the linear fit, where the slope (0.18) indicates the proportional relationship between both methods, and the intercept (0.19) reflects a baseline offset. The shaded green region denotes the 95 % confidence interval, and black error bars represent measurement uncertainties for each technique.

Fig. 5 shows the linear fit, confidence interval, and correlation obtained from total mean concentrations of each element in this study using both analytical techniques. The overall comparison between the two methods gave an R^2^ of 0.85, beta (95 % CI = 0.11 [0.077, 0.29]), and F value of 29.11. Pearson’s correlation was conducted between ICP-MS and benchtop XRF measured trace elements gave *r* = 0.95 at *p* ≤ 0.05 confidence level with corresponding (slope, intercept) of (0.18, 0.19). The black error bars illustrate the measurement uncertainties for both methods. The red line shows a strong linear correlation (R^2^ = 0.85) between ICP-MS and benchtop XRF measurements, meaning 85 % of the benchtop XRF measurements can be predicted by the ICP-MS results (Fig. 5).

### Limits of agreement and mean bias between the methods (Bland-Altman analysis)

**Fig. 6.**
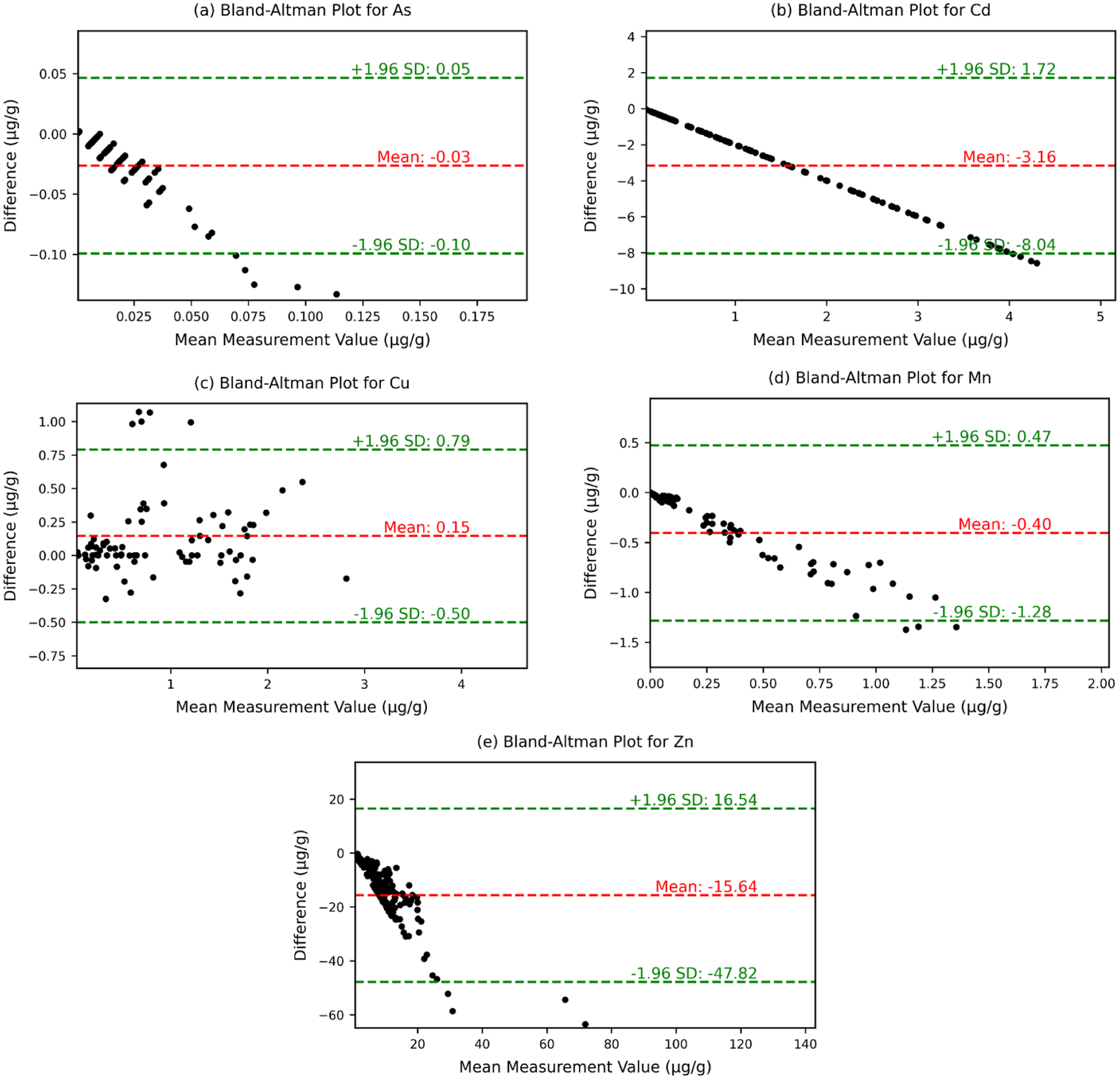
Bland-Altman plots illustrating the differences in elemental concentrations (μg/g) measured by benchtop XRF and ICP-MS for (a) As, (b) Cd, (c) Cu, (d) Mn, and (e) Zn. The x-axis represents the mean concentration measured by both techniques, while the y-axis displays their difference. The red dashed line denotes the mean bias between methods, with the green dashed lines marking the 95 % limits of agreement (±1.96 standard deviations).

The Bland-Altman analysis for trace elements in rat tissues provides a comparison of ICP-MS and benchtop XRF methods by calculating the mean bias and the limits of agreement at a 95 % confidence level. The limits of agreement are determined as the mean bias ±1.96 times the standard deviation (SD) of the differences between the two methods. This interval represents the range within which 95 % of the differences between the methods are expected to fall, indicating how interchangeable the two methods are (Fig. 6) [64].

Cd has a mean difference of −3.16 μg/g, with limits of agreement spanning from −8.04 μg/g to 1.72 μg/g. This range shows moderate agreement between the two methods for Cd. As demonstrates a very small mean difference of −0.03 μg/g, and narrow limits of agreement from −0.10 μg/g to 0.05 μg/g, indicating high consistency between the two methods. For Zn, the analysis shows a mean difference of −15.64 μg/g with limits of agreement ranging from −47.82 μg/g to 16.54 μg/g, indicating some variability between the methods. Mn exhibits a smaller mean difference of −0.40 μg/g and tighter limits of agreement from −1.28 μg/g to 0.47 μg/g, suggesting better concordance. Lastly, Cu shows a mean difference of 0.15 μg/g, with limits of agreement from −0.50 μg/g to 0.79 μg/g, which still reflects a reasonable level of consistency. Overall, the analysis reveals that benchtop XRF provides comparable results to ICP-MS, particularly for As, Cu and Mn, where the agreement is highest. These findings support the potential interchangeability of the two methods within the specified limits of agreement.

### Effect of tissue weights on trace elemental concentrations

Our tissue weights (grams) ranged from 0.008 – 0.76 g (grams) with a mean value of 0.31 g. The ANOVA model identifying differences in elemental content based on weights gave F statistics of 1.563 (*p* = 0.18) Thus, these results did not show any significant difference between the metal concentrations and sample weights. Furthermore, Pearson’s correlation identifying relationships between the metal concentrations and sample weights found only copper to be significant (*p* < 0.01) from both the concentrations of copper with ICP-MS and benchtop XRF (correlation coefficients of 0.38 and 0.31; *p* < 0.01) respectively.

## Discussion

To the best of our knowledge, this study presents the first attempt to assess and evaluate the potential of a benchtop XRF device as a method to quantify elemental contents in a wide variety of tissues simultaneously. Previous feasibility and validity studies relating to the assessment of metal and metalloid concentrations in tissues and biological samples including human liver, bones, toenails, have predominantly focused on portable XRF technology validated through ICP-MS [23,25,30–32,34,47,65]. The benchtop XRF demonstrated detection limits spanning from 0.004 μg/g for Cd to 0.350 μg/g for Mn in tissue samples, showcasing its capability to detect trace elements with high precision in complex biological matrices. A comparison with a prior study using the same benchtop XRF for toenail analysis provides additional context for the system’s performance [21]. For instance, while the toenail study reported detection limits of 0.12 μg/g for Cu and 0.18 μg/g for Zn, our study achieved detection limits of 0.040 μg/g for Cu and 0.120 μg/g for Zn, demonstrating the robustness of the benchtop XRF in analyzing tissue samples.

While calibration is commonly performed using doped reference standards, the XRF methodology employed in this study utilizes actual biological tissues as calibration materials. However, the slightly low calibration R^2^ values for some elements, such as Cd with 0.86, Mn with 0.87, and Zn with 0.80, suggest limitations in the tissue sample’s calibration accuracy. These values indicate that the calibration models may not have perfectly captured the variability in elemental concentrations across different tissue samples. The lower calibration R^2^ values could be due to the limited range of reference standards used in calibration, as evidenced by the narrow concentration ranges for each element. For instance, the reference range for Cd was only from 0.090 to 0.100 μg/g, which is quite narrow and might not extensively cover the broader spectrum of concentrations in all tissue samples. This restricts the model’s ability to predict values outside this narrow range accurately. To address these discrepancies and improve the calibration’s predictive power, it is recommended to use a broader range of calibration standards that more comprehensively represent the expected variations in sample compositions. This approach will enhance the calibration’s robustness, allowing for more accurate predictions across a wider array of sample types and concentrations.

The Bland-Altman analysis for trace elements illustrates the agreement between ICP-MS and benchtop XRF methods. Using *a* ± 1.96 SD range, this analysis reveals that while some elements show greater variability, elements like Mn and As exhibit narrow limits of agreement, indicating high reliability, and Cd shows moderate agreement, underscoring the benchtop XRF’s consistency with ICP-MS for these elements. The Bland-Altman analysis further corroborates these observations, offering valuable insights into the agreement between the two methods. The mean biases for As (−0.03 μg/g), Cu (0.15 μg/g), and Mn (−0.40 μg/g) were all close to zero, indicating good agreement between ICP-MS and BXRF for these elements. For As, the small mean bias and narrow limits of agreement suggest minimal systematic error and a high degree of precision in measurements obtained using benchtop XRF when compared to ICP-MS. Cu and Mn also showed consistent agreement, as evidenced by their small mean biases and tighter clustering of differences around the zero-difference line. In contrast, Cd and Zn showed larger biases (−3.16 μg/g and −15.64 μg/g, respectively), because the broader range of differences likely stems from the intrinsic low concentrations of these elements in biological tissues and the variability in detection limits. For Cd, the issue may stem from its low concentrations in some tissues, while Zn’s challenges could be attributed to spectral overlaps and its relatively wide range of concentrations across samples. These results emphasize that while benchtop XRF performs well to quantify certain trace elements; its performance remains element-dependent and is influenced by both instrumentation sensitivity and matrix-specific factors.

In terms of measurement precision, our study achieved lower CV for Mn (1.30 %) and Cd (2.18 %) compared to the toenail study with the benchtop XRF, which reported CVs of 3.73 % and 16.67 %, respectively for the same elements. These findings emphasize the reliability of benchtop XRF in delivering consistent results across different biological matrices and underscore its adaptability for both tissue and keratinized sample analyses. Similarly, our obtained CV values for Cu and Zn were around the range of values reported in a study of human liver tissues (3.2 % and 1.0 % respectively) measured using portable XRF spectrometry. [34] In another study, the CV values for ICP-MS that measured inorganic elemental concentrations in human tissue samples were generally higher than those obtained in this present study: for As (0.86 μM), Cu (158 μM), Mn (1.7 μM), and Zn (144 μM), and the CVs were 9.9 %, 4.0 %, 2.7 %, and 4.4 %, respectively. In contrast, our present study showed CVs of 1.71 % for As, 1.01 % for Cu, 1.30 % for Mn, and 0.22 % for Zn, indicating that the benchtop XRF method showed better precision [13].

We examined the relationship between tissue weights and trace elemental concentrations, the absence of a significant difference as indicated by the ANOVA model suggested that the amount of metal detected is not strongly dependent on the sample size. This means that for most of the trace elements analyzed, their concentration per unit mass does not change drastically with the sample’s mass in the given range. The lack of significance in the model coefficients implies that there is no consistent trend across the samples linking weight to elemental concentration, which agrees with past approaches utilizing a similar normalization approach to reduce dependence on mass [21,25]. In a situation like this, if tissue weight significantly impacted trace element concentrations, we would expect to see a clear and consistent trend as the sample mass changes. In addition to this, the Pearson correlation coefficients showed a slight but significant relationship between tissue weight and Cu concentrations measured by both ICP-MS and benchtop XRF methods. This could be due to Cu’s biological role or distribution within the tissue types sampled. However, there would be no reason to think that both ICP-MS and XRF would have a strong dependence on Cu concentrations by weight, but not other elemental concentrations by weight. The collective findings indicate that variations in sample tissue weights do not significantly impact the elemental concentration results obtained through the utilization of both analytical instruments employed in this study. This is significant for XRF, as no sample preparation was done to standardize the sample weight prior to measurement, but the measurement relied on a normalization approach using the Compton scattering from the anode to identify corrections for weight and geometry. This further confirms that the calibration technique is valuable in further reducing any preparation prior to measurement and can reduce potential matrix effects through multiple tissues or organs measured.

The findings from this study have highlighted both the strengths and limitations of benchtop XRF compared to ICP-MS for elemental analysis in biological tissues. The lower sensitivity of BXRF for certain elements became evident when considering their performance in correlation and regression analyses. While the R^2^ values for As, Mn, and Cd remained strong, the lower R^2^ values for Cu and Zn suggest that BXRF was less effective at capturing these elements accurately. This discrepancy is likely due to BXRF’s limited sensitivity for elements present at low concentrations or within complex biological matrices. Technical challenges, such as signal interference from neighboring elements and matrix effects specific to biological tissues, are known issues that could have further impacted the accuracy of Cu and Zn quantifications. For instance, Zn often exhibits overlapping spectral peaks with other elements, complicating its detection. To address these limitations, the study suggests several improvements. First, the inclusion of a wider range of calibration standards with varying elemental concentrations would enhance BXRF’s calibration model. This approach would allow for more accurate predictions across a broader spectrum of tissue types and concentrations, mitigating the issue of limited calibration ranges observed in this study. Additionally, extending the measurement duration beyond 7.5 min or increasing the X-ray tube power such as from 15 watts to 50 watts could significantly reduce detection limits that would bring about a better quantification of low-concentration elements. Importantly, such advancements could be achieved without sacrificing the non-destructive and high-throughput nature of BXRF, making it a more viable alternative to ICP-MS. The Epsilon 4 benchtop XRF system used in this study offers unique advantages over portable XRF systems [23,32,42,65–70]. Operating at 50 kV and 15 watts, the benchtop system delivers enhanced detection limits and higher precision through slightly prolonged measurement times. While the measurement duration of 7.5 min per sample may seem lengthy, it is significantly faster than the time-intensive processes associated with ICP-MS, which require extensive sample preparation, including digestion and dilution. The ability to perform simultaneous multi-elemental quantification with minimal operator expertise further solidifies BXRF’s practicality for routine analyses at a time when large quantity is to be measured. The system’s robust calibration approach, leveraging Compton normalization to account for variations in tissue mass and geometry, further underscores its potential for reliable measurements in diverse biological matrices.

Furthermore, our findings drew attention to the nuances and interplay between the two methodologies. We observed that while both technologies hold their unique advantages, certain constraints of the ICP-MS method, particularly relating to dilution increasing uncertainty, were evident in the data. For instance, the noise in lower concentrations of the ICP-MS results suggest a detection limit issue, where the sensitivity of the method is hampered by the dilution of tissue samples. This phenomenon was especially noticeable with elements like As and Cd, where the measurements obtained through XRF did not display similar dispersion patterns. The dilution inherent to ICP-MS can lead to a loss of detection for samples with both low mass and concentrations, highlighting one of the method’s limitations. In contrast to this, the benchtop XRF with its non-destructive sample analysis does not necessitate sample dilution or have this dependency on mass for low-level detection. For the purpose of practicability, it becomes clear that the choice of method should be made with consideration of a particular study’s needs, the sample’s nature, and result requirements. Where BXRF shines with its speed and non-destructive analysis, ICP-MS offers superior sensitivity in most samples of adequate weight.

To further refine benchtop XRF quantification, particularly for elements like Zn that present challenges due to spectral interferences and matrix effects, several targeted methodological enhancements can be explored. One promising approach is the use of multi-energy excitation settings tailored to amplify element-specific fluorescence signals. By fine-tuning the excitation conditions, Zn detection could be significantly improved without unnecessarily extending acquisition times or introducing unwanted background noise. Advancements in detector technology also offer a pathway to improved precision. The integration of ultra-low noise silicon drift detectors (SDDs) with enhanced energy resolution could sharpen peak differentiation, mitigating spectral overlaps and boosting signal-to-noise ratios. This would be particularly beneficial for low-abundance elements that struggle with detection thresholds. Beyond hardware improvements, adaptive spectral fitting techniques could be employed to dynamically adjust for tissue-specific attenuation effects. By accounting for subtle variations in sample composition, these real-time corrections would enhance the accuracy of elemental quantification, reducing systematic biases that often arise in heterogeneous biological matrices. A more targeted refinement would be selectively extending measurement times only for elements with inherently weak fluorescence signals rather than uniformly increasing scan durations across all analytes. This strategic adjustment would enhance sensitivity where needed without compromising overall efficiency, ensuring that benchtop XRF remains a practical, high-throughput tool for biological tissue analysis.

Most importantly, the translational implications of this study are substantial, particularly for human tissue and clinical applications. While prior studies employing portable XRF have demonstrated its feasibility for measuring metals like As, Pb, and Mn in select biological matrices, the benchtop XRF expands this capability to include tissues of varying thicknesses and weights. Its enhanced sensitivity and precision make it well-suited for applications where accurate quantification of trace metals and metalloids is critical, such as in toxicology, epidemiology, and clinical diagnostics. For instance, quantifying trace metals in tissues can provide critical insights into health conditions linked to metal exposures, such as Pb toxicity, Cd-induced kidney damage, or Mn-related neurodegeneration [71–73]. By enabling rapid, non-destructive, and accurate analyses, benchtop XRF holds the potential to transform how elemental measurements are conducted in both research and clinical settings. Moreover, large-scale and routine elemental quantification could become a reality with the widespread adoption of BXRF, as it eliminates the need for labor-intensive procedures and expensive consumables required by ICP-MS. This capability could empower researchers and clinicians to monitor environmental exposures, assess health risks, and develop targeted interventions for populations affected by metal pollution, such as Pb from battery disposals or Cd from industrial waste. The ability to accurately measure both low and high concentrations of metals and metalloids in biological tissues is critical for advancing our understanding of their roles in health and disease [18,74]. To this course, the benchtop XRF with its unique combination of speed, accuracy, and versatility, is poised to play a pivotal role in addressing these challenges and advancing the field of elemental analysis [21].

This study underscores the potential for incorporating benchtop XRF into human tissue analyses, leveraging its robustness and sophistication to address the limitations of other elemental measurement techniques. The benchtop XRF system employed in this study offers significant advantages over portable XRF devices, which, while convenient for field applications, lack the precision and throughput capabilities necessary for high-quality biological research and clinical diagnostics. The benchtop XRF system’s ability to analyze up to ten samples simultaneously in batch mode significantly increases efficiency, making it particularly advantageous for large-scale studies or high-throughput clinical workflows. Furthermore, the system’s inherent deconvolution algorithms and normalization processes, such as Compton scattering correction, ensure consistent and reliable results across tissues of varying thicknesses, densities, and compositions. This capability is especially critical when translating the method to human studies, where the heterogeneity of biological matrices is more pronounced than in controlled animal models. The reliability demonstrated in this study, validated against the gold standard ICP-MS, highlights the benchtop XRF’s potential for accurately quantifying trace metals and metalloids in human tissues which are vital for understanding environmental exposures and associated health outcomes. Translation of these findings to human studies will also involve optimizing the benchtop XRF system for clinical workflows. This includes reducing measurement time while maintaining sensitivity and ensuring that the system is adaptable for routine use in healthcare settings. The non-destructive nature of BXRF makes it an ideal candidate for applications requiring repeated measurements, such as measuring essential elements that are critical for metabolic and immune functions (e.g., Cu and Zn), assessing nutritional deficiencies or imbalances (e.g., Zn), tracking toxic metal accumulation (e.g., As or Cd), and neurological and renal diseases (e.g., Pb or Cd) over time, which could become a standard practice in clinical and epidemiological settings. By bridging the gap between animal studies and human clinical applications, this research positions benchtop XRF as a pivotal tool for advancing precision health and understanding environmental metal exposures, while also providing a scalable, efficient, and accurate solution for elemental analysis, facilitating its integration into environmental, toxicological, and clinical research. Moreover, integration with advanced computational techniques, such as machine learning for spectral deconvolution, may improve accuracy and streamline the analysis of complex biological matrices. The scalability of BXRF to large-scale human studies holds significant promise for its use in population-based research, occupational health, and clinical diagnostics.

In summary, the results of this study demonstrate that while benchtop XRF may not achieve the same sensitivity as ICP-MS for many elements, it can provide equally precise results for detecting trace elements in biological tissues, particularly for low-mass samples, with a quick 7.5-minute measurement time and detection limits spanning from 0.004 to 0.350 μg/g. The Bland-Altman analysis, performed to assess the agreement between the two methods, demonstrated strong concordance, with particularly notable reliability observed for As, Cu, and Mn. Given the simplicity and rapidness of BXRF techniques, this method offers considerable time and cost savings over ICP-MS, which requires a more laborious and consumable intensive preparation process. Future efforts should also investigate its potential in pediatric applications, where sample preservation and minimal invasiveness are crucial. Therefore, the integration of benchtop XRF into human studies has the potential to revolutionize routine elemental measurement, facilitating better understanding, diagnosis, and monitoring of health conditions linked to trace element exposures and deficiencies.

## Figures and Tables

**Table 1:** Goodness of fit parameters for benchtop XRF calibrations for tissue-based reference materials. The RMSE of the calibration, range of reference standards, coefficients of variation, and limit of detection (LOD) are given in μg/g dry weight.

Benchtop XRF (μg/g, d.w)	System Calibration (R^2^)	Mean detection value (μg/g)*	Coefficient of Variation (%)	Root mean square error (μg/g)	Range of reference standards (μg/g)	
As	0.91	7.16±0.120	1.71	0.1200	6.910	7.400
Cd	0.86	0.097±0.0021	2.18	0.0002	0.090	0.100
Cu	0.89	1.99±0.0201	1.01	0.0202	1.960	2.040
Mn	0.87	13.51±0.180	1.30	0.1800	13.13	13.80
Zn	0.80	24.68±0.059	0.22	0.0520	24.68	24.88

* represent mean ± standard deviation and d.w means dry weight.

**Table 2:** Comparative detection limits and total elemental concentrations measured by benchtop XRF and ICP-MS analyses evaluated across all tissues.

BXRF (μg/g)	Total elemental mean (μg/g)*	Limit of detection (μg/g)
As	0.0085±0.012	0.250
Cd	0.0038±0.0037	0.004
Cu	0.97±0.6900	0.040
Mn	0.19±0.2100	0.350
Zn	3.86±7.0091	0.120
ICP-MS (μg/g)	Total elemental mean (μg/g)*	Limit of detection (μg/g)
As	0.035±0.048	0.382
Cd	3.16±2.49	0.264
Cu	0.808±0.66	1.147
Mn	0.5895±0.64	0.605
Zn	19.50±22.031	0.627

* represent mean ± standard deviation; LOD for BXRF was determined using 30 repeated measurements on two randomly selected, cleaned rat tissue samples. It was calculated as twice the standard deviation of measured concentrations, normalized by the regression slope between X-ray fluorescence counts and elemental concentrations, representing the instrument’s response factor. The LOD for ICP-MS was determined by first calculating the square root of the raw intensities measured during the rinse phase, which were then divided by the net counts of the actual sample. This ratio was subsequently multiplied by the concentration of the sample and its relative standard deviation (RSD).

## Data Availability

Data will be made available on request.

## References

[1] Navas-Acien A, Schwartz BS, Rothenberg SJ, Hu H, Silbergeld EK, Guallar E, Bone lead levels and blood pressure endpoints, Epidemiology. (Fairfax) 19 (3) (2008) 496–504, 10.1097/EDE.0b013e31816a2400. 18414090

[2] Weisskopf MG, Proctor SP, Wright RO, Schwartz J, Spiro A, Sparrow D, , Cumulative lead exposure and cognitive performance among elderly men, Epidemiology. (Fairfax) 18 (1) (2007) 59–66, 10.1097/01.ede.0000248237.35363.29. 17130688

[3] Navas-Acien A, Guallar E, Silbergeld EK, Rothenberg SJ, Lead exposure and cardiovascular disease—A systematic review, Environ. Health Perspect 115 (3) (2007) 472–482, 10.1289/ehp.9785. 17431501 PMC1849948

[4] Weuve J, Press DZ, Grodstein F, Wright RO, Hu H, Weisskopf MG, Cumulative exposure to lead and cognition in persons with Parkinson’s disease, Movement Disorders 28 (2) (2013) 176–182, 10.1002/mds.25247. 23143985 PMC3581753

[5] Oyedele OO, Olaniyan DS, Gabriel JA, Abejide FH, Adesina KE, Assessing the environmental risk of heavy metals in surrounding areas of Lafarge Cement Industry in Shagamu, Ogun State, Nigeria, Chem. Environ. Sci. Archive 03 (02) (2023) 40–47, 10.47587/CESA.2023.3204.

[6] National Research Council. Mineral Tolerance of Animals.; 2005.

[7] Failla ML, Trace elements and host defense: recent advances and continuing challenges, J. Nutr 133 (5) (2003) 1443S–1447S, 10.1093/jn/133.5.1443S. 12730439

[8] Bath GS Enzootic icterus – a form of chronic copper poisoning. J. S. Afr. Vet. Assoc. 50:3–13. 551182

[9] Van Loggerenberg DE, Laver PN, Myburgh JG, Botha CJ, Diagnostic value of energy dispersive hand-held X-ray fluorescence spectrometry in determining trace element concentrations in ovine liver, Biol. Trace Elem. Res 190 (2) (2019) 358–361, 10.1007/s12011-018-1546-0. 30315508

[10] Takahashi S, Takahashi I, Sato H, Kubota Y, Yoshida S, Muramatsu Y, Determination of major and trace elements in the liver of Wistar rats by inductively coupled plasma-atomic emission spectrometry and mass spectrometry, Lab. Anim 34 (1) (2000) 97–105, 10.1258/002367700780577966. 10759373

[11] Laur N, Kinscherf R, Pomytkin K, Kaiser L, Knes O, Deigner HP, ICP-MS trace element analysis in serum and whole blood, PLoS. One 15 (5) (2020), 10.1371/journal.pone.0233357. PMC7239469 32433650

[12] Ishii C, Nakayama SMM, Kataba A, Ikenaka Y, Saito K, Watanabe Y, , Characterization and imaging of lead distribution in bones of lead-exposed birds by ICP-MS and LA-ICP-MS, Chemosphere 212 (2018) 994–1001, 10.1016/j.chemosphere.2018.08.149. 30286556

[13] Grassin-Delyle S, Martin M, Hamzaoui O, Lamy E, Jayle C, Sage E, , A high-resolution ICP-MS method for the determination of 38 inorganic elements in human whole blood, urine, hair and tissues after microwave digestion, Talanta 199 (2019) 228–237, 10.1016/j.talanta.2019.02.068. 30952251

[14] Nunes JA, Batista BL, Rodrigues JL, Caldas NM, Neto JAG, Barbosa F, A simple method based on ICP-MS for estimation of background levels of arsenic, cadmium, copper, manganese, nickel, lead, and selenium in blood of the Brazilian population, J. Toxicol. Environ. Health a 73 (13–14) (2010) 878–887, 10.1080/15287391003744807. 20563921

[15] Mohd-Taufek N, Cartwright D, Davies M, Hewavitharana AK, Koorts P, Shaw PN, , The simultaneous analysis of eight essential trace elements in Human milk by ICP-MS, Food Anal. Methods 9 (7) (2016) 2068–2075, 10.1007/s12161-015-0396-z.

[16] McIntosh KG, Guimarães D, Cusack MJ, Vershinin A, Chen ZW, Yang K, , Evaluation of portable XRF instrumentation for assessing potential environmental exposure to toxic elements, Int. J. Environ. Anal. Chem 96 (1) (2016) 15–37, 10.1080/03067319.2015.1114104. 33746339 PMC7978405

[17] Specht AJ, Obrycki JF, Mazumdar M, Weisskopf MG, Feasibility of lead exposure assessment in blood spots using energy-dispersive X-ray fluorescence, Environ. Sci. Technol 55 (8) (2021) 5050–5055, 10.1021/acs.est.0c06622. 33759507 PMC10615324

[18] Adesina KE, Burgos CJ, Grier TR, Sayam ASM, Specht AJ, Ways to measure metals: from ICP-MS to XRF, Curr. Environ. Health Rep 12 (1) (2025) 7, 10.1007/s40572-025-00473-y. 39865194 PMC11913532

[19] Perring L, Andrey D, ED-XRF as a tool for rapid minerals control in milk-based products, J. Agric. Food Chem 51 (15) (2003) 4207–4212, 10.1021/jf034158p. 12848486

[20] Mantler M, Schreiner M, X-ray fluorescence spectrometry in art and archaeology, X-Ray Spectrometry 29 (1) (2000) 3–17, 10.1002/(SICI)1097-4539(200001/02)29:1<3::AID-XRS398>3.0.CO;2-O.

[21] Specht AJ, Adesina KE, Read DE, Weisskopf MG, Benchtop x-ray fluorescence to quantify elemental content in nails non-destructively, Sci. Total Environ 918 (2024) 170601, 10.1016/j.scitotenv.2024.170601. 38309346 PMC10923075

[22] Bhatia M, Specht AJ, Ramya V, Sulaiman D, Konda M, Balcom P, , Portable X-ray fluorescence as a rapid determination tool to detect parts per million levels of Ni, Zn, As, Se, and Pb in Human toenails: a South India case study, Environ. Sci. Technol (2021), 10.1021/acs.est.1c00937. Published online September 16,:acs.est.1c00937. PMC8582015 34529917

[23] Specht AJ, Weisskopf M, Nie LH, Portable XRF technology to quantify Pb in bone *In vivo*, J. Biomark 2014 (2014) 1–9, 10.1155/2014/398032. PMC4437356 26317033

[24] Specht AJ, Obrycki JF, Mazumdar M, Weisskopf MG, Feasibility of lead exposure assessment in blood spots using energy-dispersive X-ray fluorescence, Environ. Sci. Technol 55 (8) (2021) 5050–5055, 10.1021/acs.est.0c06622. 33759507 PMC10615324

[25] Specht AJ, Kponee K, Nkpaa KW, Balcom PH, Weuve J, Nie LH, , Validation of x-ray fluorescence measurements of metals in toenail clippings against inductively coupled plasma mass spectrometry in a Nigerian population, Physiol. Meas 39 (8) (2018) 085007, 10.1088/1361-6579/aad947. 30091720 PMC6156793

[26] Kinoshita H, Tanaka N, Jamal M, Kumihashi M, Okuzono R, Tsutsui K, , Application of energy dispersive X-ray fluorescence spectrometry (EDX) in a case of methomyl ingestion, Forensic Sci. Int 227 (1–3) (2013) 103–105, 10.1016/j.forsciint.2012.08.026. 22999231

[27] Watanabe H, Determination of trace metals in water using x-ray fluorescence spectrometry, Talanta 19 (11) (1972) 1363–1375, 10.1016/0039-9140(72)80133-4. 18961191

[28] Stump IG, Carruthers J, D’Auria JM, Applegarth DA, F Davidson AG, Quantitative analysis of trace elements in human blood and plasma by energy dispersive X-ray fluorescence, Clin. Biochem 10 (1977) 127–132, 10.1016/S0009-9120(77)91740-4. 884840

[29] Specht AJ, Zhang X, Young A, Nguyen VT, Christiani DC, Ceballos DM, , Validation of in vivo toenail measurements of manganese and mercury using a portable X-ray fluorescence device, J. Expo Sci. Environ. Epidemiol 32 (3) (2022) 427–433, 10.1038/s41370-021-00358-w. 34211112 PMC8720103

[30] Specht AJ, Mostafaei F, Lin Y, Xu J, Nie LH, Measurements of strontium levels in Human bone In vivo using portable X-ray fluorescence (XRF), Appl. Spectrosc 71 (8) (2017) 1962–1968, 10.1177/0003702817694383. 28756702 PMC5617116

[31] Hampton JO, Specht AJ, Pay JM, Pokras MA, Bengsen AJ, Portable X-ray fluorescence for bone lead measurements of Australian eagles, Sci. Total Environ 789 (2021) 147998, 10.1016/j.scitotenv.2021.147998. 34051503

[32] Zhang X, Specht AJ, Wells E, Weisskopf MG, Weuve J, Nie LH, Evaluation of a portable XRF device for in vivo quantification of lead in bone among a US population, Sci. Total Environ 753 (2021) 142351, 10.1016/j.scitotenv.2020.142351. 33207470 PMC7677595

[33] Bueno Guerra MB, de Almeida E, Carvalho GGA, Souza PF, Nunes LC, Júnior DS, , Comparison of analytical performance of benchtop and handheld energy dispersive X-ray fluorescence systems for the direct analysis of plant materials, J. Anal. At. Spectrom 29 (9) (2014) 1667–1674, 10.1039/C4JA00083H.

[34] Milman N, Laursen J, Podenphant J, Asnaes S, Trace elements in normal and cirrhotic human liver tissue I. Iron, copper, zinc, selenium, manganese, titanium and lead measured by X-ray fluorescence spectrometry, Liver. 6 (2) (1986) 111–117, 10.1111/j.1600-0676.1986.tb00276.x. 3736354

[35] Borjesson J, Barregard L, Sallsten G, Schutz A, Jonson R, Alpsten M, , In vivo XRF analysis of mercury: the relation between concentrations in the kidney and the urine, Phys. Med. Biol 40 (3) (1995) 413–426, 10.1088/0031-9155/40/3/006. 7732071

[36] Barregård L, Svalander C, Schütz A, Westberg G, Sällsten G, Blohmé I, , Cadmium, mercury, and lead in kidney cortex of the general Swedish population: a study of biopsies from living kidney donors, Environ. Health Perspect 107 (11) (1999) 867–871, 10.1289/ehp.107-1566723. 10544153 PMC1566723

[37] Groskopf C, Bennett SR, Gherase MR, Fleming DEB, Detection of lead in bone phantoms and arsenic in soft tissue phantoms using synchrotron radiation and a portable x-ray fluorescence system, Physiol. Meas 38 (2) (2017) 374–386, 10.1088/1361-6579/aa513f. 28134135

[38] Chettle DR, In vivo applications of X-ray fluorescence in human subjects, Pramana 76 (2) (2011) 249–259, 10.1007/s12043-011-0038-y.

[39] Börjesson J, Mattsson S, In vivo x-ray fluorescence measurements of lead, cadmium and mercury in occupational and environmental studies: a review of work conducted in Sweden 1970–2005, X-Ray Spectrometry 37 (1) (2008) 58–68, 10.1002/xrs.995.

[40] Boerjesson J, Studies of cadmium, mercury and lead in man, The Value of X-Ray Fluores. Measure. in Vivo (1996).

[41] Börjesson J, Mattsson S, Alpsten M, Trace element concentrations studied in vivo using X-ray fluorescence analysis, Applied Radiat. Isotopes 49 (5–6) (1998) 437–445, 10.1016/S0969-8043(97)00264-9. 9569512

[42] Zhang X, Specht AJ, Weisskopf MG, Weuve J, Nie LH, Quantification of manganese and mercury in toenail in vivo using portable X-ray fluorescence (XRF), Biomarkers 23 (2) (2018) 154–160, 10.1080/1354750X.2017.1380082. 28901783 PMC5987198

[43] Ahmid K, Specht A, Morikawa L, Ceballos D, Wylie S, Lead and other toxic metals in plastic play foods: results from testing citizen science, lead detection tools in childcare settings, J. Environ. Manage 321 (2022) 115904, 10.1016/j.jenvman.2022.115904. 36104879

[44] Specht AJ, Kirchner KE, Weisskopf MG, Pokras MA, Lead exposure biomarkers in the Common loon, Sci. Total Environ 647 (2019) 639–644, 10.1016/j.scitotenv.2018.08.043. 30092519 PMC6168339

[45] Kim R, Aro A, Rotnitzky A, Amarasiriwardena C, Hu H, K X-ray fluorescence measurements of bone lead concentration: the analysis of low-level data, Phys. Med. Biol 40 (9) (1995) 1475–1485, 10.1088/0031-9155/40/9/007. 8532760

[46] Whitcomb BW, Schisterman EF, Assays with lower detection limits: implications for epidemiological investigations, Paediatr. Perinat. Epidemiol 22 (6) (2008) 597–602, 10.1111/j.1365-3016.2008.00969.x. 19000298 PMC2723785

[47] Specht AJ, Dickerson AS, Weisskopf MG, Comparison of bone lead measured via portable x-ray fluorescence across and within bones, Environ. Res 172 (2019) 273–278, 10.1016/j.envres.2019.02.031. 30822560 PMC6511307

[48] U.S. Public Health Service. Agency for Toxic Substances and Disease Registry, Toxicological Profile for Arsenic.; 1989. 37184170

[49] U.S. Public Health Service. Agency for Toxic Substances and Disease Registry, Toxicological Profile for Cadmium.; 1989. 24049863

[50] Suzuki KT, Kubota K, Takenaka S, Copper in cadmium-exposed rat kidney metallothionein, Chem. Pharm. Bull. (Tokyo) 25 (10) (1977) 2792–2794, 10.1248/cpb.25.2792. 589716

[51] Schroeder HA, Nason AP, Interactions of trace metals in rat tissues. Cadmium and nickel with zinc, chromium, copper, manganese, J. Nutr 104 (2) (1974) 167–178, 10.1093/jn/104.2.167. 4810977

[52] Julshamn K, Utne F, Braekkan OR, Interactions of cadmium with copper, zinc and iron in different organs and tissues of the rat, Acta Pharmacol. Toxicol. (Copenh) 41 (5) (2009) 515–524, 10.1111/j.1600-0773.1977.tb02163.x. 579562

[53] Kägi Jeremias H. R., Nordberg Monica, eds. Experientia Supplementum. In: Metallothionein.; 1978.

[54] Shankar AH, Prasad AS, Zinc and immune function: the biological basis of altered resistance to infection, Am. J. Clin. Nutr 68 (2) (1998) 447S–463S, 10.1093/ajcn/68.2.447S. 9701160

[55] Bubb JM, Lester JN, The impact of heavy metals on lowland rivers and the implications for man and the environment, Sci. Total Environ 100 (1991) 207–233, 10.1016/0048-9697(91)90379-S. 2063183

[56] Bisgård KM, Laursen J, Nielsen BS, Energy-dispersive XRF spectrometry using secondary radiation in a cartesian geometry, X-Ray Spectrometry 10 (1) (1981) 17–24, 10.1002/xrs.1300100106.

[57] ValkoviC V, Analysis of Biological Material For Trace Elements Using X-Ray Spectroscopy, CRCpress Inc, 1980.

[58] Woldseth R, X-Ray Energy Spectrometry, Kevex Corp., 1973.

[59] Daniel MacDougall, Crummett WB, , Guidelines for data acquisition and data quality evaluation in environmental chemistry, Anal. Chem 52 (14) (1980) 2242–2249, 10.1021/ac50064a004.

[60] Han FX, Patterson WD, Xia Y, Sridhar BBM, Su Y, Rapid determination of mercury in plant and soil samples using inductively coupled plasma atomic emission spectroscopy, a comparative study, Water. Air. Soil. Pollut 170 (1–4) (2006) 161–171, 10.1007/s11270-006-3003-5.

[61] Kim R, Aro A, Rotnitzky A, Amarasiriwardena C, Hu H, K X-ray fluorescence measurements of bone lead concentration: the analysis of low-level data, Phys. Med. Biol 40 (9) (1995) 1475–1485, 10.1088/0031-9155/40/9/007. 8532760

[62] Zaki R, Bulgiba A, Ismail R, Ismail NA, Statistical methods used to test for agreement of medical instruments measuring continuous variables in method comparison studies: a systematic review, PLoS. One 7 (5) (2012) e37908, 10.1371/journal.pone.0037908. 22662248 PMC3360667

[63] Martin Bland J, Altman DouglasG, Statistical methods for assessing agreement between two methods of clinical measurement, The Lancet 327 (8476) (1986) 307–310, 10.1016/S0140-6736(86)90837-8. 2868172

[64] Giavarina D, Understanding Bland Altman analysis, Biochem. Med. (Zagreb) 25 (2) (2015) 141–151, 10.11613/BM.2015.015. 26110027 PMC4470095

[65] Specht AJ, Zhang X, Goodman BD, Maher E, Weisskopf MG, Nie LH, A dosimetry study of portable X-ray fluorescence in vivo metal measurements, Health Phys. 116 (5) (2019) 590–598, 10.1097/HP.0000000000000971. 30624351 PMC6433501

[66] Kalnicky DJ, Singhvi R, Field portable XRF analysis of environmental samples, J. Hazard. Mater 83 (1–2) (2001) 93–122, 10.1016/S0304-3894(00)00330-7. 11267748

[67] Fleming DEB, Crook SL, Evans CT, Nader MN, Atia M, Hicks JMT, , Portable X-ray fluorescence of zinc applied to human toenail clippings, Journal of Trace Elements in Medicine and Biology 62 (2020) 126603, 10.1016/j.jtemb.2020.126603. 32623095

[68] McIntosh KG, Guimarães D, Cusack MJ, Vershinin A, Chen ZW, Yang K, , Evaluation of portable XRF instrumentation for assessing potential environmental exposure to toxic elements, Int. J. Environ. Anal. Chem 96 (1) (2016) 15–37, 10.1080/03067319.2015.1114104. 33746339 PMC7978405

[69] Specht AJ, Steadman DW, Davis M, Bartell SM, Weisskopf MG, Bone lead variability in bone repository skeletal samples measured with portable x-ray fluorescence, Sci. Total Environ 880 (2023) 163197, 10.1016/j.scitotenv.2023.163197. 37001655 PMC13372475

[70] McIver DJ, VanLeeuwen JA, Knafla AL, Campbell JA, Alexander KM, Gherase MR, , Evaluation of a novel portable x-ray fluorescence screening tool for detection of arsenic exposure, Physiol. Meas 36 (12) (2015) 2443–2459, 10.1088/0967-3334/36/12/2443. 26536141

[71] Eiró LG, Ferreira MKM, Frazão DR, Aragão WAB, Souza-Rodrigues RD, Fagundes NCF, , Lead exposure and its association with neurological damage: systematic review and meta-analysis, Environ. Sci. Pollut. Res 28 (28) (2021) 37001–37015, 10.1007/s11356-021-13536-y. 34046839

[72] Liu C, Ju R, Manganese-induced neuronal apoptosis: new insights into the role of endoplasmic reticulum stress in regulating autophagy-related proteins, Toxicolog. Sci 191 (2) (2023) 193–200, 10.1093/toxsci/kfac130. 36519822

[73] Yan LJ, Allen DC, Cadmium-induced kidney injury: oxidative damage as a unifying mechanism, Biomolecules. 11 (11) (2021) 1575, 10.3390/biom11111575. 34827573 PMC8615899

[74] Adesina KE, Espejo W, Celis JE, Sandoval M, Specht AJ, First report of metals and metalloids on bone and claw tissues of Humboldt penguins (Spheniscus humboldti), Austral J Vet Sci 56 (3) (2024) 135–140, 10.4206/ajvs.563.03.

